# Somatostatin receptor staining in FFPE sections using a ligand derivative dye as an alternative to immunostaining

**DOI:** 10.1371/journal.pone.0172030

**Published:** 2017-02-09

**Authors:** Koki Hasegawa, Shinji Kudoh, Takaaki Ito

**Affiliations:** 1 Department of Pathology and Experimental Medicine, Kumamoto University, Graduate School of Medical Sciences, Kumamoto, Japan; 2 Center for Instrumental Analysis, Kyoto Pharmaceutical University, Kyoto, Japan; University of Minnesota Medical Center, UNITED STATES

## Abstract

The confirmation of target expression in tissues is a prerequisite for molecular-targeted therapy. However, difficulties are sometimes associated with the production of appropriate antibodies against receptors. We herein developed a ligand derivative dye for the staining of receptors. The somatostatin receptor (sstr) was selected as the target and FITC-octreotate as the detective agent. We performed a blot analysis to detect sstr in the transfer membrane. The sstr2 recombinant protein or cell lysate from a small cell lung carcinoma cell line (H69) was boiled and loaded onto SDS-PAGE, and the proteins were transferred to a membrane. Even after denaturing processes, FITC-octreotate still bound sstr on the membrane. Furthermore, FITC-octreotate depicted the expression of sstr in formalin-fixed and paraffin-embedded (FFPE) sections, a method that we named ligand derivative staining (LDS). The accuracies of immunostaining and LDS were compared at the points of the detection of sstr using FFPE sections of 30 neuroendocrine tumor specimens. The sensitivity of LDS was 81.8%, while those of immunostaining using anti-sstr2 and sstr5 antibodies were 72.7% and 63.6%, respectively. Thus, LDS appears to be superior to immunostaining. A ligand derivative may be used as a substitute for antibodies, and has the potential to support economical, simple, and accurate detection methods.

## Introduction

Antibodies have been applied to a large number of biological assays and therapies. In recent years, the confirmation of target molecule expression in cancer tissues has become a prerequisite, particularly in molecular target therapy. Immunohistochemistry (IHC) is one of the most widely used techniques. Polyclonal antibodies, which are harvested from animal serum, vary among animals and difficulties are associated with quality control. Monoclonal antibodies are produced from immortalized cells and theoretically have the same quality. However, it has not yet been determined whether these cells produce exactly the same antibodies after several years [1]. Regarding the production of antibodies for membrane proteins, challenges are associated with the design of epitopes because membrane proteins often form multimer and extracellular domains that are tightly packed. Therefore, epitopes may be masked from the cell surface. Moreover, the utilization of receptors as immunogens is often demanding because of the difficulties in expressing and purifying receptors [2]. Another reason is the accessibility of an antibody to an epitope. In order to disrupt the masking of an epitope, some antigen retrieval techniques have been developed [3]. However, additional work is needed in order to select the appropriate antigen retrieval technique for each antibody. For example, G-protein-coupled receptors (GPCRs) are major constituents of cell-surface receptors. A large number of studies have reported that many GPCRs exist as dimers or multi-oligomers. Some of these studies have also shown that the multimerization of GPCRs is important for their functions, including ligand-binding affinity, potency, and efficacy [4]. Some GPCRs also form sodium dodecyl sulphate (SDS)-resistant complexes [5]. Therefore, we demonstrated that the somatostatin receptor (sstr), which belongs to the family of GPCRs, maintains its ligand-binding ability after SDS polyacrylamide gel electrophoresis (SDS-PAGE) and transferal onto a blotting membrane.

Formalin-fixed and paraffin-embedded (FFPE) tissues, which are suitable for maintaining tissue morphology and the long-term fixing of their molecules, are used in pathology laboratories. The success of staining for a specific substance in archival FFPE tissues linked with clinical records allows retrospective findings in a cohort study. Many proteins in FFPE tissues may be denatured during the formalin fixation and paraffin-embedding processes. However, a large number of membrane proteins may be resistant to a certain degree to denaturing conditions and maintain some of their functions such as ligand binding. Therefore, we herein performed staining for a specific receptor using its ligand as the receptor recognition agent instead of antibodies.

The actions of sstr are mediated by six receptor subtypes: sstr1, sstr2a, sstr2b, sstr3, sstr4, and sstr5. These sstr subtypes were previously reported to be constantly present in neuroendocrine tumors [6]. Tumor imaging and peptide receptor radionuclide therapy (PPRT) using radiolabeled somatostatin analogues such as OctreoScan or ^68^Ga-DOTA-TOC are now widespread. Therefore, sstr is established diagnostic and therapeutic importance as a molecular target. In order to further develop these imaging techniques or therapies, the determination of sstr expression in individual patients is an important prerequisite [7]. Recent studies on sstr IHC demonstrated that the monoclonal antibody, anti-sstr2 [UMB-1], was adequate for reliability. The accuracy of IHC results was confirmed by a comparison with *in vitro* ^125^I-[Tyr_3_]-octreotide autoradiography. According to Körner M et al. [8], tumor tissue sections with highly autoradiographic intensities exhibited 86% staining with an anti-sstr2 [UMB-1] antibody. This finding indicates that detection accuracy is not always coincident between the antibody (IHC) and peptide ligand (autoradiography). Regarding the technical aspect of IHC, antibodies are utilized as specific probes and have the ability to bind the target molecule in tissue sections. Focusing on receptors as molecular targets, most receptors originally possess binding abilities to their ligands; therefore, we were prompted to use ligands instead of antibodies as specific recognition and strongly binding molecules.

Most archival pathological specimens are generally fixed with formalin and embedded in paraffin in order to prevent autolysis and preserve morphology. The application of FFPE sections is needed to pursue the universalization of detection with ligand derivatives. Although information regarding the nature of proteins in FFPE tissues is limited, previous studies showed that protein folding was maintained even after fixation with formaldehyde [9–10]. In the present study, we demonstrated that the receptor maintained its ligand-binding ability following the denaturing treatment. After SDS-PAGE, proteins were transferred to a nitrocellulose membrane and the target receptor was specifically stained by its ligand, which was named a western ligand blot (WLB). We then applied the ligand derivative dye in order to visualize the localization of the target receptor in FFPE sections, and called this ligand derivative staining (LDS). LDS using FITC-octreotate was compared with IHC using anti-sstr2 and anti-sstr5 antibodies. Detection sensitivities were compared between LDS and IHC using FFPE neuroendocrine tumor sections from 30 patients.

## Materials and methods

### The synthesis of FITC-β-Ala-PEG_2_-Suc-octreotate (FITC-octreotate)

NH_2_-β-Ala-PEG_2_-Suc-D-Phe-Cys(Trt)-Tyr(tBu)-D-Trp(Boc)-Lys(Boc)-Thr(tBu)-Cys(Trt)-Thr(tBu) was assembled from HMPB-ChemMatrix resin (Biotage, Uppsala, Sweden) with the standard 9-fluorenyl-methyloxycarbonyl (Fmoc) protocol using Fmoc-amino acid derivatives (Peptide Institute, Osaka, Japan) and Fmoc-PEG_2_-Suc-OH (AnaSpec, Fremont, CA). A mixture of FITC (Thermo Scientific, Waltham, MA) and DIPEA in DMF was added to the protected peptide resin and reacted for 1 hour. The resin obtained was washed with methanol and dried *in vacuo*. An aliquot of the resin was treated with a mixture of TFA/water/ethanedithiol/triisopropylsilane (94:2.5:2.5:1) for 2 hours, followed by precipitation in cold dimethyl ether. The crude peptide was purified by HPLC on a 5C_18_ ARII column (4.6 × 250 mm; Nacalai Tesque, Kyoto, Japan) with two solvent systems and the gradient elution method at a flow rate of 1 mL/min. Solvent A was 0.1% TFA in water, and solvent B was 0.1% TFA in acetonitrile. After purification, a disulfide bond was formed by the air oxidation method. Briefly, FITC-β-Ala-PEG_2_-Suc-[^2,7^Cys(SH)]octreotate (1 mg) was dissolved in phosphate-buffered saline (3 mL), and DMSO (0.1 mL) was added. After 15 hours, FITC-β-Ala-PEG_2_-Suc-octreotate, which was referred to as FITC-octreotate, was purified under the same conditions as those described above. After lyophilization, FITC-octreotate was obtained as a yellow powder. FITC-octreotate was measured by matrix-assisted laser desorption/ionization-time of flight mass spectroscopy. The calculated mass for (M+H)^+^ was 1740.96. The detected mass for (M+H)^+^ was 1741.35. The concentration analysis of FITC-octreotate was quantified using ultraviolet-visible spectrophotometry (ε = 93,000, λ = 490 nm).

### Determination of the binding affinity of FITC-octreotate for sstr2

The binding affinity profile of FITC-octreotate was determined by using the SSTR2 Human Recombinant Protein (Abnova, Taiwan). A 96-well microplate (Thermo Fisher scientific, Roskilde, Denmark) was coated with 50 μL (210 ng) of SSTR2 Human Recombinant Protein in 100 mM NaHCO_3_ solution at 4°C for 20 h. The plate was washed twice with Tris buffer. The wells were blocked for 2 h with 200 μL of blocking buffer (20% blocking one (Nacalai tesque, Kyoto, Japan) in deionized water). Then, appropriate dilutions (from 10,000 nM to 0.01 nM of FITC-octreotate) were incubated in the wells at 25°C for 1 h. After incubation, the plates were washed five times with TBST buffer (20 mM Tris—HCl [pH 7.5], 150 mM NaCl, and 0.1% Tween 20). Anti-FITC antibody (Dako, Denmark) at a dilution of 1:250 in 5% blocking one in TBS buffer was added to each well and incubated at 25°C for 1 h. After incubation, the plates were washed five times with TBST. TMB solution (Nacalai tesque, Kyoto, Japan) was added to each well and incubated at 25°C for 1 h. Reaction was terminated by adding 0.1M H_2_SO_4_ solution. The absorbance in each well was measured in a microplate reader Infinite 200 (TECAN, Männedorf, Switzerland). The graph was plotted with absorbance on the ordinate and each different concentration on the abscissa. Kd value was calculated from curve fitting model using KaleidaGraph software.

### Double staining analysis

A human small cell lung cancer cell line (H69) cells (1 x 10^5^) were washed twice with 50 mM Phosphate buffered saline (PBS) and suspended in PBS (200μL). H69 cells suspension was added to the well of cytospin device and spun for 5 minutes (500 rpm). The attached cells on the slide were fixed with 95% ethanol for 30 minutes. After washing twice with PBS, the cells were treated with 5% Triton X-100 in PBS for 5 minutes. After washing twice with PBS, the cells were blocked with 1% Block Ace (Bio-Rad Laboratories, Hercules, CA) in PBS for 15 minutes. The cells were incubated with the mixture of rabbit anti-sstr2 (ab134152, Abcam, Cambridge, England, diluted 1:100) antibody and FITC-Octreotate (10 μM) in antibody diluent (DAKO, Glostrup, Denmark) at 4°C overnight. After washing twice with PBS, the cells were incubated with goat anti-rabbit IgG conjugated with Alexa 568 (Thermo Fisher scientific, Waltham, MA, diluted 1:400) for 1 hour. After washing twice with PBS, DAPI (Sigma-Aldrich, St. Louis, MO, diluted 1:1000) in PBS was incubated for 15 minutes. The cells were mounted with Fluoromount / Plus (Diagnostic BioSystems, Pleasanton, CA). Fluorescent images were observed with microscope IX71 (Olympus, Tokyo, japan).

### Cell lysis

A human small cell lung cancer cell line (H69) was purchased from the American Type Cell Collection (Manassas, VA). H69 cells were grown on RPMI 1640 medium; supplemented with 2 mM L-glutamine, 10 nM HEPES, 1 mM sodium pyruvate, 4.5 g/l glucose, 1.5 g/l sodium bicarbonate, and 20% FBS and incubated at 37°C in 5% CO_2_ and saturated humidity. Cells were lysed on ice in 150 mM NaCl, 1% Triton X-100, 0.5% sodium deoxycholate, 0.1% SDS, 1 mM EDTA-2Na (pH 8.0), 1 mM EGTA (pH 7.5), 2.5 mM sodium pyrophosphate, 1 mM b-glycerophosphate, 1 mM Na_2_VO_4_, 1 mM PMSF, and 20 mM Tris-HCl (pH 7.5) containing a protease inhibitor cocktail (Roche Diagnostics, Basel, Switzerland). Protein concentrations were determined using the BCA assay (Bio-Rad Laboratories, Hercules, CA).

### SDS-PAGE

Samples were heated at 95°C for 5 min with Laemmli buffer. Equal amounts of protein were electrophoresed on polyacrylamide gels containing 0.1% SDS. Proteins on the gel were transferred onto a Hybond ECL nitrocellulose membrane (GE Healthcare, Fairfield, CT) in a mini transblot apparatus (Atto, Tokyo, Japan) at 120 V for 1 hour using Tris—glycine buffer (48 mM Tris, 39 mM glycine, 0.037% SDS, and 20% methanol at pH 9.2).

### Amido black stain

Transferred membrane was rinsed H_2_O for 5 minutes. The membrane was dipped in the solution containing 0.1% Amido black, 50% methanol and 10% acetic acid for 1 minute. The membrane was washed with the solution containing 90% methanol and 2% acetic acid for 5 minutes.

### Western blotting

After blocking the membrane at room temperature for 1 hour with 5% skim milk in TBST buffer (20 mM Tris—HCl [pH 7.5], 150 mM NaCl, and 0.1% Tween 20), the blot was probed with rabbit anti-somatostatin receptor 2 (UMB-1) and 5 (UMB-4) monoclonal antibodies (ab134152, ab109495, Abcam, Cambridge, England) at a dilution of 1:1000 in 5% skim milk in TBST buffer at 4°C for 13 hours. This was followed by a 1-hour incubation with a horse anti-rabbit IgG antibody conjugated with HRP (GE Healthcare) at a dilution of 1:10000 in 5% skim milk in TBST buffer. Regarding the detection of the protein signal, the ECL Prime western blotting detection system (GE Healthcare) was used according to the manufacturer’s instructions.

### Western ligand blotting

After blocking the membrane at room temperature for 1 hour with 5% skim milk in TBST buffer, the blot was probed with FITC-octreotate at 0.1 nM in 5% skim milk in TBST buffer at 4°C for 13 hours. After 15-minutes washing thrice in TBST buffer, this was followed by a 1-hour incubation in a rabbit anti-fluorescein antibody conjugated with HRP (ab19492, Abcam) at a dilution of 1:10000 in 5% skim milk in TBST buffer at room temperature. In order to detect the protein signal, the ECL Prime western blotting detection system (GE Healthcare) was used according to the manufacturer’s instructions. In the blocking study, octreotide was added at 1 μM to the step of the reaction with FITC-octreotate.

### Tissue specimens

Thirty surgically resected neuroendocrine tumors from various organs were obtained at Kumamoto University Hospital and Japanese Red Cross Kumamoto Hospital (Table 1). The samples were fixed with 10% formalin and embedded in paraffin. Tissue sections were stained with HE. A histological diagnosis of the samples was made according to the criteria of the World Health Organization [11–12]. Additional sections were used for IHC staining. This study was approved by the Ethics Board at Kumamoto University and Japanese Red Cross Kumamoto hospital. All experiments were carried out in accordance with the approved guidelines and regulations. All specimens obtained from patients with general consent were collected according to the guidelines of use of pathology archives (approval number 1091).

**Table 1:** Detection status of sstr in cases with neuroendocrine tumor.

Case No	Location of the investigated tumor	SSTR2a	SSTR5	Octreotate
1	lung	-	-	3
2	lung	-	-	3
3	rectum	2	3	3
4	sigmoid colon	3	-	3
5	stomach	1	-	3
6	stomach	3	3	3
7	jejunum	3	-	3
8	duodenum	-	3	1
9	rectum	2	1	-
10	lung	-	1	3
11	rectum	2	3	3
12	duodenum	3	3	3
13	rectum	2	3	3
14	rectum	-	-	-
15	rectum	-	-	-
16	rectum	-	-	1
17	duodenum	3	3	3
18	rectum	-	-	-
19	duodenum	2	3	3
20	rectum	3	-	-
21	rectum	-	-	-
22	rectum	3	-	-
23	rectum	2	2	1
24	rectum	-	-	-
25	sigmoid colon	-	-	-
26	duodenum	3	3	3
27	rectum	-	-	-
28	rectum	-	3	-
29	rectum	-	-	-
30	stomach	2	3	1

### IHC

Three-micrometer-thick sections were cut from a paraffin block and placed on coated glass slides (Matsunami Glass, Osaka, Japan). Sections were deparaffinized with xylene, and rehydrated in graded ethanol. Endogenous peroxidase activity was quenched by incubating with 0.3% hydrogen peroxide-methanol for 10 min. Antigen retrieval was subsequently performed with microwave irradiation at 95°C in 10 mM citrate buffer for 20 min. After blocking using Protein Block, Serum-Free (DAKO, Glostrup, Denmark) for 5 min, the sections were incubated with the rabbit anti-sstr2 (diluted1:100) or anti-sstr5 (diluted1:100) antibody at 4°C overnight. After washing three times with TBST, sections were incubated with a secondary antibody: anti-rabbit IgG conjugated with HRP (Histofine Simple Stain MAX PO, Nichirei Co., Tokyo, Japan) at room temperature for 30 min. After washing three times with TBST, reaction products were visualized by the Liquid DAB+ Substrate Chromogen System (DAKO). Nuclei were counterstained with hematoxylin.

### LDS

The deparaffinization and rehydration steps of the sections were performed using the same procedure described above. After blocking using Protein Block, Serum-Free (Dako) for 5 min, sections were incubated with FITC-octreotate (200 nM) in antibody dilution reagent (Dako) at room temperature for 5 min. After washing three times with TBST, the sections were reacted with the amplification reagent, Tyramide Signal Amplification (CSA II, Dako). FITC-octreotate was detected by an anti-fluorescein antibody conjugated with HRP (DAKO) by incubating for 15 min. After washing three times with TBST, the amplification solution including fluorescein-labeled tyramide was incubated in the dark for 5 min. Amplified fluorescein was detected using an anti-fluorescein antibody conjugated with HRP (DAKO) and incubating for 15 min. After washing three times with TBST, reaction products were visualized by the Liquid DAB+ Substrate Chromogen System (DAKO). Nuclei were counterstained with hematoxylin.

### Analysis of IHC and LDS

IHC and LDS results were semiquantitatively assessed. Stained areas were scored in light microscopy in 10 different fields under a magnification of 200X. The expression of sstr was graded on a three-tiered system. The proportion of the stained tumor cell area was expressed in percentages with increments of 25 (i.e., negative cases had less than 25% of the tumor cell stained area as -; positive cells had 26% to 50% as 1+; 51% to 75% as 2+; and 76% to 100% as 3+).

## Results

### Synthesis of FITC-octreotate

**Fig 1:**
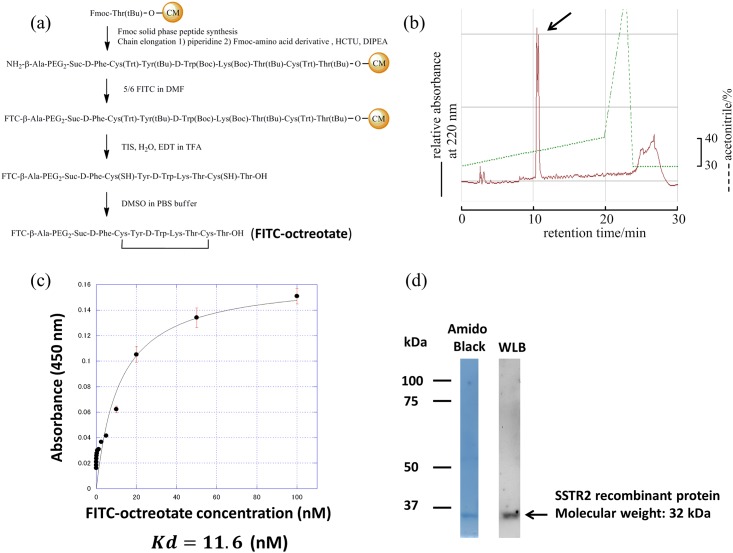
Synthesis of FITC-octreotate and evaluation of affinity. Synthetic route for FITC-octreotate. (a) FITC-octreotate was synthesized using the Fmoc solid phase method from ChemMatrix resin (CM) attached to the first amino acid. β-Ala and PEG2-Suc were introduced as linkers at the amino group after peptide chain elongation. FITC was modified at the N terminus. The protected peptide resin was treated with TFA. A disulfide bond was formed by the air oxidation method. (b) The RP-HPLC elution profile of FITC-octreotate. Column: Cosmosil 5C18 ARII (4.6 X 250mm) (Nacalai Tesque. Kyoto. Japan): eluent: 0.1% TFA in aqueous acetonitrile using the linear gradient indicated on the chromatogram flow rate: 1 mL/min. The arrow indicates FITC-octreotate. The product obtained was the isomer of FITC-octreotate caused by using the 5/6-FITC mixed isomer. (c) Saturation binding assay using FITC-octreotate on Somatostatin receptor subtype 2a. (d) Amido black stain and WLB membranes transferring sstr2 recombinant protein after SDS-PAGE.

Octreotate was synthesized starting from ChemMatrix (CM) resin using a 9-fluorenyl-methyloxycarbonyl (Fmoc) solid-phase peptide synthesis protocol (Fig 1a). After elongation, FITC was conjugated with the N-terminus. The protected peptide resin was treated with trifluoroacetic acid (TFA). A disulfide bond was formed by air oxidation and the crude peptide was purified with reverse phase high-performance liquid chromatography (RP-HPLC) (Fig 1b). FITC-octreotate was obtained as a yellow powder after lyophilization. The compound was measured by matrix-assisted laser desorption/ionization-time of flight mass spectroscopy.

### Determination of binding affinity of FITC-octreotate for sstr2 recombinant protein

The affinity of FITC-octreotate was determined by Enzyme-Linked Immuno Sorbent Assay (ELISA). Sstr2 human recombinant protein was coated onto the wells of microplates. Appropriate dilutions (from 5 nM to 0.01 nM of FITC-octreotate) were incubated with sstr2. After incubation, FITC was detected by anti-FITC antibody conjugated by HRP. A 3,3',5,5'-tetramethylbenzidine (TMB) solution is added and is converted by the HRP to a color signal. The graph was plotted with absorbance on the ordinate and each different concentration on the abscissa. Kd value was calculated from curve fitting model. As the result, Kd value was determined 16 nM. (Fig 1c)

### Determination of binding ability of FITC-octreotate for sstr2 recombinant protein after denaturing process

Sstr2 recombinant protein was performed SDS-PAGE and transferred to the nitrocellulose membrane. The membrane was stained by Amido black to visualize one specific band corresponding to sstr2 recombinant protein (Fig 1d, lane Amido black). On the other hand, the membrane was analyzed by WLB at reaction concentration at 0.1 nM FITC-octreotate to visualize one specific band corresponding to the same position in CBB-stained membrane (Fig 1d, lane WLB).

### Double staining analysis with anti-sstr2 antibody and FITC-octreotate

**Fig 2:**
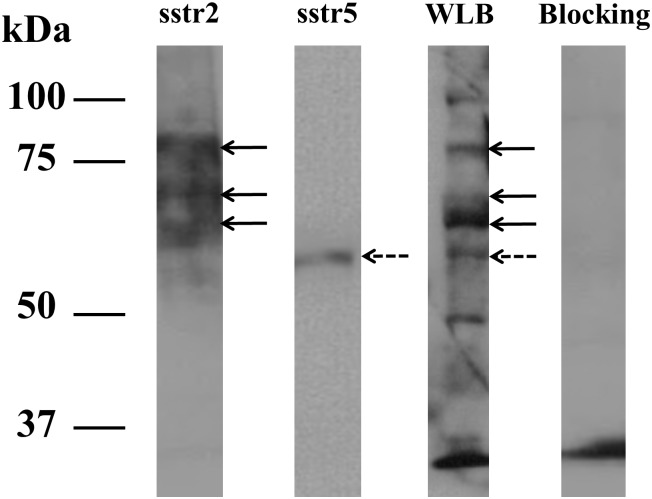
Double stain analysis. Double stain analysis with FITC-octreotate and anti-sstr2a antibody. Immunofluorescence image of H69 cells on the slide were stained with DAPI (blue), FITC-octreotate (green) and Alexa 568-anti-sstrR2a antibody (red). The solid arrows indicate the double-stained cells with FITC-octreotate and anti-sstr2a antibody.

H69 cells on the slide was stained with anti-sstr2 antibody and FITC-octreotate. Anti-sstr2 antibody was visualized by Alexa 568 in membranous staining (Fig 2, anti-sstr2, red). In contrast, FITC-octreotate was visualized by FITC in membranous and cytoplasmic staining (Fig 2, FITC-octreotate, green). Merged image showed the anti-sstr2 antibody and FITC-octreotate could specifically bind to H69 cells.

### Comparison of western blots using anti-sstr2 and anti-sstr5 antibodies with WLB using FITC-octreotate

**Fig 3:**
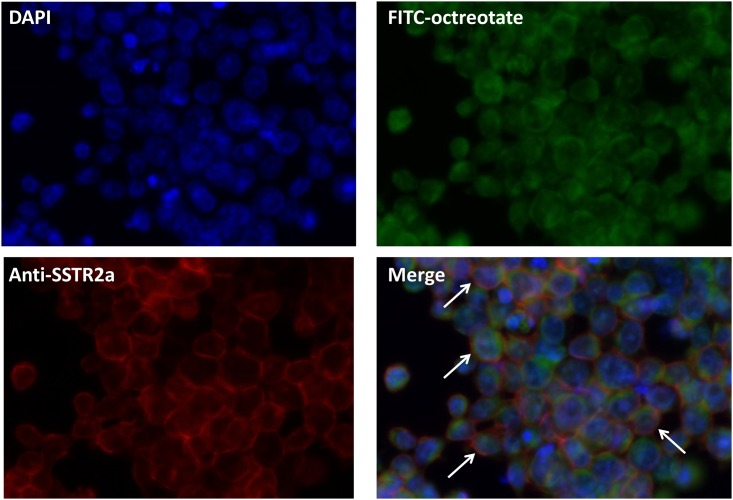
WB and WLB analysis. A Western blot analysis of the somatostatin receptor using anti-sstr2 (lane sstr2) and anti-sstr5 (lane sstr5) antibodies and a Western ligand blot analysis with FITC-octreotate (lane WLB) of a small-cell lung cancer cell (H69) lysate. All experiments were performed under the same conditions. The locations of the bands in the western blot analysis (the solid arrows of lane sstr2 and the dashed arrow of lane sstr5) correspond approximately to those in the western ligand blot analysis (the solid arrows and dashed arrow of lane WLB). The bands in the western ligand blot analysis (the solid arrows and dashed arrow of lane WLB) were completely blocked by the addition of a large amount of octreotide (X10000) (lane Blocking).

Western blot analyses of a sstr-expressing small cell lung carcinoma cell line (H69) lysate using the anti-sstr2 antibody [UMB-1] yielded three specific bands (Fig 3, lane sstr2), while that using the anti-sstr5 antibody [UMB-4] yielded a single band (Fig 3, lane sstr5). Corresponding to these bands, four bands were yielded in the WLB analysis with FITC-octreotate (Fig 3, lane WLB). The bands in the WLB analysis were completely blocked by the addition of octreotide as the blocking peptide (Fig 3, lane Blocking). The specificity of FITC-octreotate for the detection of sstr was demonstrated because lanes sstr2 and sstr5 closely corresponded to lane WLB. Octreotate has moderate affinity for sstr3. The band appeared in approximately 50kDa (Fig 3, lane WLB) is estimated sstr3 (Predicted molecular weight: 46 kDa). The band appeared in approximately 100kDa (Fig 3, lane WLB) is estimated the receptor dimer.

### Comparison of LDS using FITC-octreotate with IHC using anti-sstr2 and anti-sstr5 antibodies in FFPE sections

**Fig 4:**
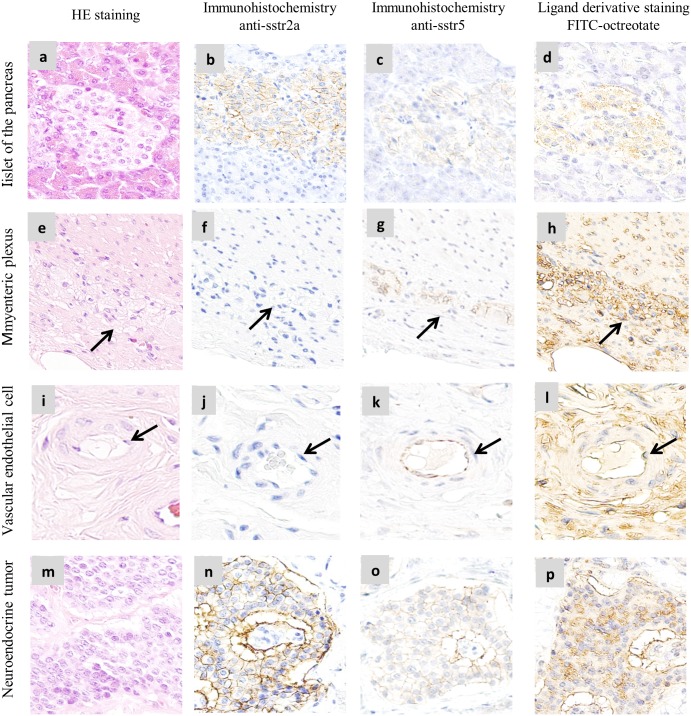
Comparison of IHC with LDS using FFPE sections. Comparison of immunohistochemistry using anti-sstr2 (b, f, j, n) and anti-sstr5 (c, g, k, o) antibodies with ligand derivative staining using FITC-octreotate (d, h, l, p) in myenteric plexuses, vascular endothelial cells, islets of the pancreas, and neuroendocrine tumors. Corresponding tissues were also stained by HE (a, e, i, m). All FFPE tissue sections were cut at a thickness of 3 μm from human pathological samples. The arrows (e, f, g, h) indicate myenteric plexuses and arrows (i, j, k, l) indicate vascular endothelial cells.

Pancreatic islet and myenteric plexus cells, which were previously reported to constantly express sstr, were selected as positive control cells [13–14]. Pancreatic islet cells were stained using the anti-sstr2 and anti-sstr5 antibodies (Fig 4b and 4c). In contrast, the myenteric plexus was stained using the anti-sstr5 antibody only (Fig 4f). Growing vascular endothelium cells, in which sstr was previously shown to be preferentially expressed, were partially stained by the anti-sstr5 antibody (Fig 4k) [15]. Neuroendocrine tumors were stained by the anti-sstr2 and anti-sstr5 antibodies (Fig 4n and 4o). In comparison with these results, the corresponding cells were stained by LDS using FITC-octreotate at reaction concentration 200 nM approximated antibody concentration in immunohistochemistry (Fig 4d, 4h, 4l and 4p). The corresponding tissues were also stained by hematoxylin and eosin (HE) (Fig 4a, 4e, 4i and 4m).

### Comparison of detection sensitivities between LDS and IHC in neuroendocrine tumor sections from 30 patients

Thirty cases of neuroendocrine tumors, which often express sstr, were stained in an attempt to evaluate the usefulness of LDS for FFPE sections (Table 1). IHC with anti-sstr2 was positive in 16 sections (53.3%) while that with anti-sstr5 was positive in 14 sections (46.7%). LDS of FITC-octreotate was positive in 18 sections (60.0%). Eight triple negative sections, which were defined by negative results in IHC and LDS, were identified (26.7%). Triple negative sections may not have sstrs and were consequently excluded. Therefore, we assumed that 22 neuroendocrine tumor tissues had sstrs. Sensitivities were calculated in accordance with the number of positive section numbers divided by 22 sections. The results obtained showed that the sensitivities of anti-sstr2, anti-sstr5, and FITC-octreotate were 72.7%, 63.6%, and 81.8%, respectively. Positive immunostaining for either anti-sstr2 and/or sstr5 was detected in 19 sections, at a sensitivity of 86.4% (Table 2).

**Table 2:** Semiquantitatively evaluated detection rate and sensitivity of IHC (anti-sstr2 and sstr5) and LDS using FITC-octreotate in tumor tissues.

	SSTR2	SSTR5	LDS
Detection rate (%)*	53.3% (16/30)	46.7% (14/30)	60.0% (18/30)
Sensitivity (%)**	72.7% (16/22)	63.6% (14/22)	81.8% (18/22)
Sensitivity (%)***	86.4% (19/22)		81.8% (18/22)

* >24% stained tumor cell ratio in 30 tumor sections.

** The true positive ratio was defined as the fraction of sections in 22 positively diagnosed cases, excluded 8 triple negative cases.

*** Octreotate binds SSTR2 and 5. The immunochemical positive ratio of SSTR2, 5, or both was combined to compare the sensitivity of LDS.

## Discussion

We herein focused on the development of WLB and LDS. Detection sensitivities were compared between LDS and IHC using clinical specimens. FITC-conjugated octreotate was synthesized and determined the specific binding ability for sstr using recombinant sstr2 protein and H69 cells. We demonstrated that the ligand-binding abilities of both recombinant sstr2 and full-length sstr proteins from H69 cell and FFPE tissue were maintained after denaturing treatments including SDS-PAGE, fixation and paraffin-embedding processes. Although ligand binding in transfer membranes after SDS-PAGE and fixation tissues has been reported in some cases [16–18], conventional wisdom implies that protein functions are lost after denaturing treatments such as those using irradiating organic solutions or detergents. In contrast, we herein found that the ligand-binding ability of receptors showed resistance to denaturation. Thus, a western blot analysis of full-length sstr from H69 cell lysate revealed that the bands detected by the anti-sstr2 and anti-sstr5 antibodies. And sstr2 recombinant protein was stained by Amido black. WLB analyses also showed the same band positions corresponding to the bands detected by Amido black stain and western blot analyses.

One advantage of small molecules over antibodies is that they are chemically synthesized, which allows for a cost-effective supply, constant quality, and simple modifications to functional groups. Reaction rates may be higher because small molecules have high diffusion and infiltration rates. Reaction concentrations may also be higher due to low molecular weights. Antibodies only detect one target because of their high specificities to epitopes. However, somatostatin analogues such as octreotate have the ability to bind sstr2 and sstr5. The utilization of a similar drug derivative to the staining dye is of significant value for the accurate evaluation of drug effects. FITC-octreotate has the ability to stain the location of sstr2 and/or sstr5 in one section at one time. Furthermore, LDS employs the natural binding affinity of receptors, thereby skipping the antigen retrieval step. Therefore, the total work time needed may be shorter than that for IHC. While FITC conjugation is easily and cost-effective, the fluorescence band of fluorescein (400-500nm) is interfered with formaldehyde-induced autofluorescence from histamine and serotonin in FFPE neuroendocrine tumor section [19]. So FITC was detected by enzyme antibody technique with HRP-conjugated anti-FITC antibody and visualized the location using 3,3'-diaminobenzidine (DAB) stain.

Thirty cases of neuroendocrine tumors were stained using LDS and IHC. The results obtained showed that LDS was more sensitive than IHC, and this may be attributed to octreotate binding to sstr2 and sstr5. The detection sensitivity of IHC with anti-sstr2 was previously reported to be 86.0% [8], which is consistent with the results of the present study. In sstr-expressing tumors, the expression patterns of the subtypes differ among the tumors [20]. The most frequent subtype of expression is sstr2. However, sstr1 often predominates in prostate carcinomas and sarcomas. sstr3 is often detected in non-functioning pituitary adenomas, while sstr2 and sstr5 are frequently expressed in growth hormone-secreting pituitary adenomas. Pheochromocytoma also shows predominantly sstr3 expression and moderately sstr1, sstr2 and sstr5 expression [21, 22]. Therefore, we need to identify sstr expression patterns in each case in order to select appropriate drugs for treatments. If the LDS dye used is a molecule related to the drug being administered, drug-accumulating ability may be directly evaluated in tumor sections. We may shift from IHC to LDS in order to confirm the effects of medication on tumor tissues. In a previous study, the detection sensitivity of anti-sstr2 immunostaining was referenced to positively stained sections, which confirmed the expression of sstrs by ^125^I-[Tyr3]-octreotide autoradiography [8]. However, our results showed that three neuroendocrine tumor tissues only expressed sstr5. In order to perform accurate evaluations of drug effects in neuroendocrine tumors, the confirmation of sstr2 and sstr5 expression is preferable. LDS has the capacity to simultaneously confirm the expression of sstr2 and sstr5. Ligand staining is a simple process that does not require RI labeling and has the resolution of cell structural imaging by optical microscopy. In contrast to PCR, the presence of sstr mRNA is not always proof of the expression of sstr in tumors [23]. Since blood vessel cells also express sstr, RT-PCR may overestimate the expression of sstr in tumors [20]. Therefore, the direct staining of sstr in FFPE is of great diagnostic value.

LDS may be used to stain all archival FFPE blocks including those of rare diseases. The ligand dye has various merits and may be developed in order to evaluate a large variety of samples. In drug development, drug affinity for a target in human tissue may be assessed by LDS using FITC-modified drugs. Furthermore, FITC-modified drugs may be used as a companion diagnostic for target molecules instead of antibodies. In the present study, we developed a ligand derivative dye for the identification of drug-targeted receptors. This method is a powerful tool for studying the expression of receptors, which has not yet been well-established by the production of accurate antibodies. GPCR family members are some of the most druggable targets in human proteome research [24]. Drug accumulation for targets through GPCRs may be accurately evaluated using FFPE sections. LDS is of significant value in assessments of the effectiveness of drugs and may open new possibilities in drug development.

## References

[1] Marx V. Finding the right antibody for the job. Nature Methods.; 2013;10(8):703–7. 10.1038/nmeth.2570 23900250

[2] Hutchings CJ, Koglin M, Marshall FH. Therapeutic antibodies directed at G protein-coupled receptors. mAbs. 2010;2(6):594–606. 10.4161/mabs.2.6.13420 20864805 PMC3011214

[3] Shi S-R, Shi Y, Taylor CR. Antigen retrieval immunohistochemistry: review and future prospects in research and diagnosis over two decades. The Journal of Histochemistry and Cytochemistry. 2011;59(1):13–32. 10.1369/jhc.2010.957191 21339172 PMC3201121

[4] Bai M. Dimerization of G-protein-coupled receptors: Roles in signal transduction. Cellular Signalling. 2004;16(2):175–86. 14636888 10.1016/s0898-6568(03)00128-1

[5] Salahpour A, Angers S, Bouvier M. Functional Significance of Oligomerization of G-protein-coupled Receptors. 2000;11(5). 10.1016/s1043-2760(00)00260-5 10856916

[6] Reubi JC, Kvols LK, Waser B, Nagorney DM, Heitz PU, Charboneau JW, et al Detection of somatostatin receptors in surgical and percutaneous needle biopsy samples of carcinoids and islet cell carcinomas. Cancer Res. 1990;50(18):5969–77. 2168286

[7] Kwekkeboom DJ, Kam BL, Van Essen M, Teunissen JJM, Van Eijck CHJ, Valkema R, et al Somatostatin receptor-based imaging and therapy of gastroenteropancreatic neuroendocrine tumors. Endocrine-Related Cancer. 2010;17(1). 10.1677/ERC-09-0078 19995807

[8] Körner M, Waser B, Schonbrunn A, Perren A, Reubi JC. Somatostatin receptor subtype 2A immunohistochemistry using a new monoclonal antibody selects tumors suitable for in vivo somatostatin receptor targeting. The American journal of surgical pathology. 2012;36(2):242–52. 10.1097/PAS.0b013e31823d07f3 22251942 PMC3261429

[9] Mason JT, O’Leary TJ. Effects of formaldehyde fixation on protein secondary structure: a calorimetric and infrared spectroscopic investigation. The Journal of Histochemistry and Cytochemistry. 1991;39:225–9. 1987266 10.1177/39.2.1987266

[10] Fowler CB, Evers DL, O’Leary TJ, Mason JT. Antigen Retrieval Causes Protein Unfolding: Evidence for a Linear Epitope Model of Recovered Immunoreactivity. Journal of Histochemistry & Cytochemistry. 2011;59(4):366–81. 21411808 10.1369/0022155411400866 PMC3201144

[11] WHO Classification of Tumours of the Lung, Pleura, Thymus and Heart, 4th Edition Edited by Travis WD, Brambilla E, Burke AP, Marx A, Nicholson AG. Lyon, France, IARC, 2015 10.1097/JTO.0000000000000663 26291007

[12] Caplin ME, Baudin E, Ferolla P, Filosso P, Garcia-Yuste M, Lim E et al Pulmonary neuroendocrine (carcinoid) tumors: European Neuroendocrine Tumor Society expert consensus and recommendations for best practice for typical and atypical pulmonary carcinoids. Ann. Oncol., 26(8), 1604–1620 (2015) 10.1093/annonc/mdv041 25646366

[13] Taniyama Y, Suzuki T, Mikami Y, Moriya T, Satomi S, Sasano H. Systemic distribution of somatostatin receptor subtypes in human: an immunohistochemical study. Endocrine journal. 2005;52(5):605–11. 16284440 10.1507/endocrj.52.605

[14] Reubi JC, Laissue JA, Waser B, Steffen DL, Hipkin RW, Schonbrunn A. Immunohistochemical detection of somatostatin sst2a receptors in the lymphatic, smooth muscular, and peripheral nervous systems of the human gastrointestinal tract: Facts and artifacts. Journal of Clinical Endocrinology and Metabolism. 1999;84(8):2942–50. 10.1210/jcem.84.8.5878 10443702

[15] Watson JC, Balster D a, Gebhardt BM, O’Dorisio TM, O’Dorisio MS, Espenan GD, et al Growing vascular endothelial cells express somatostatin subtype 2 receptors. British journal of cancer. 2001;85:266–72. 10.1054/bjoc.2001.1881 11461088 PMC2364037

[16] Daniel TO, Schneider WJ, Goldstein JL, Brown MS. Visualization of lipoprotein receptors by ligand blotting. Journal of Biological Chemistry. 1983;258(7):4606–11. 6300091

[17] Salih H., Murthy G. S., Friesen H. G. Stability of hormone receptors with fixation: implications for immunocytochemical localization of receptors. Endocrinology, 105(1), 21–26 (1978) 10.1210/endo-105-1-21 221201

[18] Handa R. K. Influence of tissue fixation on the binding of ^125^I-angiotensin receptor ligands in the rat, mouse and rabbit kidney. Peptides., 23, 1847–1852 (2002) 12383873 10.1016/s0196-9781(02)00142-0

[19] Rost FWD, Ewen SWB. New methods for the histochemical demonstration of catecholamines, tryptamines, histamine and other arylethylamines by acid- and aldehyde-induced fluorescence. The Histochemical Journal. 1971; 3: 207–212. 5099266 10.1007/BF01002565

[20] Reubi JC, Waser B, Schaer JC, Laissue JA. Somatostatin receptor sst1-sst5 expression in normal and neoplastic human tissues using receptor autoradiography with subtype-selective ligands. European Journal of Nuclear Medicine. 2001;28(7):836–46. 11504080 10.1007/s002590100541

[21] Mundschenk J, Unger N, Schulz S, Höllt V, Schulz S, Steinke R, Lehnert H. Somatostatin Receptor Subtypes in Human Pheochromocytoma: Subcellular Expression Pattern and Functional Relevance for Octreotide Scintigraphy. 2016; 88 (11): 5150–5157. 10.1210/jc.2003-030262 14602742

[22] Ruggeri RM, Ferraù F, Campennì A, Simone A, Barresi V, Giuffrè G, Tuccari G, Baldari S, Trimarchi F. Immunohistochemical localization and functional characterization of somatostatin receptor subtypes in a corticotropin releasing hormone-secreting adrenal phaeochromocytoma: review of the literature and report of a case. European Journal of Histochemistry. 2009;53(1):e1. 19351607

[23] Fisher WE, Trisha A, Ii PM, Boros LG, Christopher E, Schirmer WJ. BRIEF Expression of Somatostatin. 1998;90(4):5–7.

[24] Adachi J, Kumar C, Zhang Y, Olsen JV, Mann M. The human urinary proteome contains more than 1500 proteins,including a large proportion of membrane proteins. Genome Biology, 7, R80 (2006) 10.1186/gb-2006-7-9-r80 16948836 PMC1794545

